# Plant Sterol Metabolism. Δ^7^-Sterol-C_5_-Desaturase (STE1/DWARF7), Δ^5,7^-Sterol-Δ^7^-Reductase (DWARF5) and Δ^24^-Sterol-Δ^24^-Reductase (DIMINUTO/DWARF1) Show Multiple Subcellular Localizations in *Arabidopsis thaliana* (Heynh) L

**DOI:** 10.1371/journal.pone.0056429

**Published:** 2013-02-08

**Authors:** Daniele Silvestro, Tonni Grube Andersen, Hubert Schaller, Poul Erik Jensen

**Affiliations:** 1 Department of Plant and Environmental Science, Villum Kann Rasmussen Foundation “VKR” Research Centre “Pro-Active Plants” and Center for Synthetic Biology, Faculty of Science, University of Copenhagen, Frederiksberg C, Denmark; 2 Department of Plant and Environmental Science, Center for Dynamic Molecular Interactions “DynaMo”, Faculty of Science, University of Copenhagen, Frederiksberg C, Denmark; 3 Département Réseaux Métaboliques Végétaux, Institut de Biologie Moléculaire des Plantes du CNRS, Université de Strasbourg, Strasbourg cedex, France; Lawrence Berkeley National Laboratory, United States of America

## Abstract

Sterols are crucial lipid components that regulate membrane permeability and fluidity and are the precursors of bioactive steroids. The plant sterols exist as three major forms, free sterols, steryl glycosides and steryl esters. The storage of steryl esters in lipid droplets has been shown to contribute to cellular sterol homeostasis. To further document cellular aspects of sterol biosynthesis in plants, we addressed the question of the subcellular localization of the enzymes implicated in the final steps of the post-squalene biosynthetic pathway. In order to create a clear localization map of steroidogenic enzymes in cells, the coding regions of Δ^7^-sterol-C_5_-desaturase (STE1/DWARF7), Δ^24^-sterol-Δ^24^-reductase (DIMINUTO/DWARF1) and Δ^5,7^-sterol-Δ^7^-reductase (DWARF5) were fused to the yellow fluorescent protein (YFP) and transformed into *Arabidopsis thaliana* mutant lines deficient in the corresponding enzymes. All fusion proteins were found to localize in the endoplasmic reticulum in functionally complemented plants. The results show that both Δ^5,7^-sterol-Δ^7^-reductase and Δ^24^-sterol-Δ^24^-reductase are in addition localized to the plasma membrane, whereas Δ^7^-sterol-C_5_-desaturase was clearly detected in lipid particles. These findings raise new challenging questions about the spatial and dynamic cellular organization of sterol biosynthesis in plants.

## Introduction

Sterols are well-known essential structural components that affect biophysical properties of membranes such as permeability and fluidity [1], [2] and also heat-shock tolerance [3]. Their implication in the formation of functional membrane domains together with other lipid components such as sphingolipids has been discussed [4]. Sterol derivatives are also involved in many biological processes, by acting as signalling molecules in the cell cycle [5] modulating the activity of membrane bound enzymes [6] and regulating growth since they are the precursors of steroidal hormones both in plants and animals [7], [8]. Sterol requirements in the case of plant cell division were recently studied with biosynthetic mutants; particularly, it was shown that sterol composition of the plasma membrane had an effect on the proper functioning of auxin transporters [9]. In the model plant *Arabidopsis thaliana*, campesterol is the precursor of brassinosteroids, a group of polyoxidized steroids. Their importance in plant growth and development has been demonstrated through the study of several *dwarf* mutants affected in the biosynthesis of, or in the response to, brassinosteroids. Some of these mutants are in fact deficient in the biosynthesis and accumulation of sterols which serve as brassinosteroid precursors [10]–[15]. Besides the structural and biological functions described above there is now an increasing number of reports that confer an ecophysiological relevance to sterols, as for instance in plant-pathogen interactions [16], [17] or drought stress [18].

**Figure 1:**
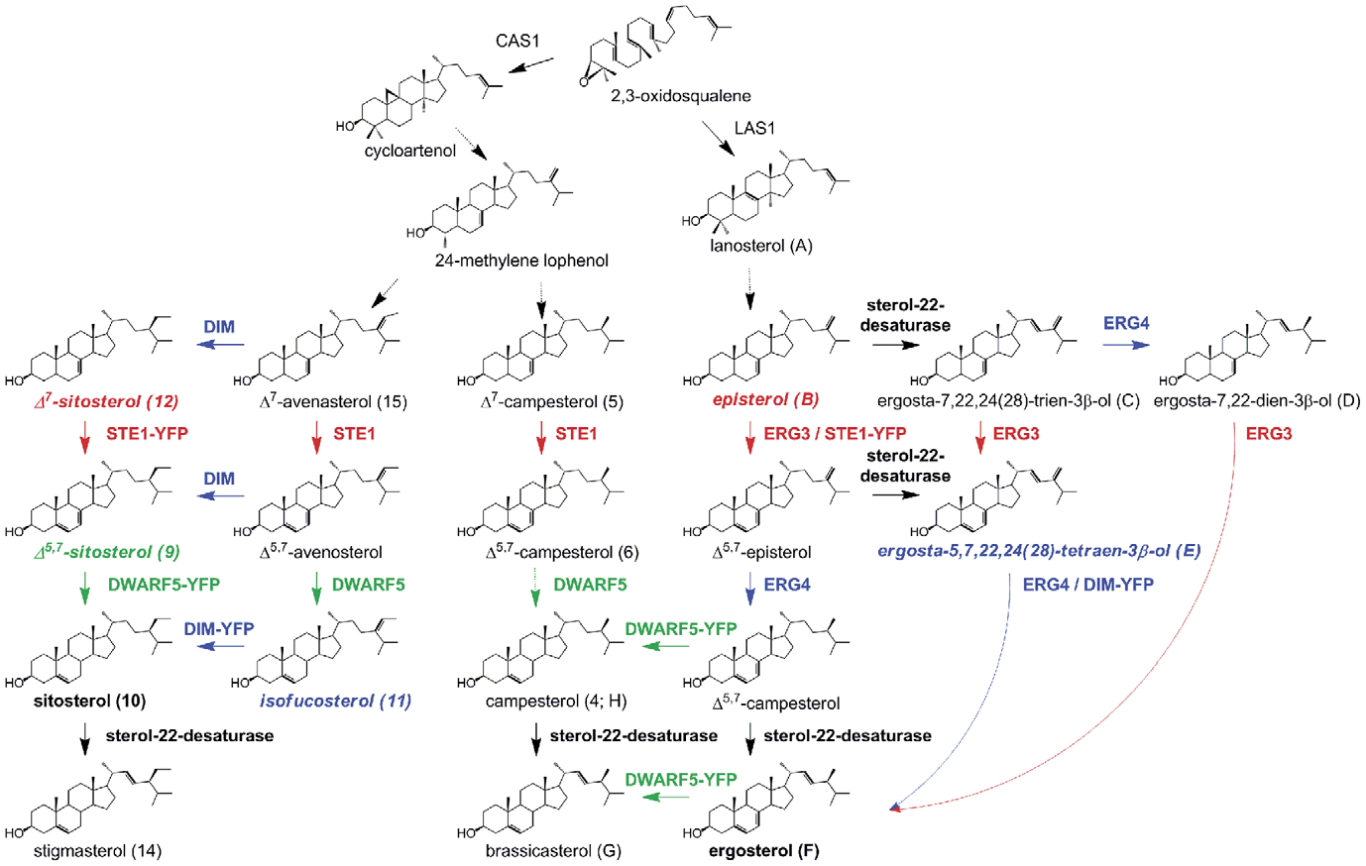
Simplified sterol biosynthetic pathway in Arabidopsis and yeast. Sterol biosynthesis starts preferentially with cycloartenol in plants and lanosterol in animals and fungi. Main biosynthetic steps and sterols accumulating in the mutant lines considered in this study are indicated in colors: red, *ste1*; green, *dwarf5*; blue, *dim1*. The major observed enzymatic activity of the YFP-fused enzymes generated is also indicated by the same color code. *LAS1* = lanosterol synthase, *CAS1* = cycloartenol synthase. The dashed arrows are indicating more than one enzymatic step. Accurate sterol nomenclature can be found at IUPAC http://www.iupac.org/

In contrast to animals, where cholesterol is the main sterol, plants accumulate a wide range of sterols with campesterol, sitosterol and stigmasterol being the major molecular species. Interestingly, a typical plant sterol profile contains little amounts of isofucosterol, the precursor of sitosterol ([19], [20]; Figure 1).In addition to 3β-hydroxy-sterols (the so-called free sterols), plants contain sterol conjugates, in particular steryl esters (SE) and steryl glycosides (SG) [21], [22]. Studies performed on a tobacco sterol overproducing mutant have shown that SE are deposited in oily droplets herein named lipid particles (LPs) [23], [24]. The low affinity of SE for membrane bilayers [25] suggested a role for these conjugates in the control of the free sterol amount in cell membranes [26]. The possible implication of the plant phospholipid sterol acyltransferase (PSAT) in this process through the enzymatically favoured esterification of sterol intermediates in the presence of high amounts of pathway end-products was recently outlined [27], [28]. Despite its importance this process is not yet clearly understood in plants.

In yeast, the LPs are well described [29] and a number of enzymes involved in sterol biosynthesis has been associated with these organelles, such as ERG1p [29], ERG6p [29], ERG7p [30] and ERG27p [31] in addition to sterol acyltransferases [32] and triacylglycerol lipases [33]. These observations strengthened the idea that the LPs operate not only as a storage organelle for lipids and sterols but also as a compartment where sterol biosynthesis occurs. So far, none of the plant sterol synthesizing enzymes has been localized to LPs. This is partly due to the general acceptance that plant sterol biosynthesis occurs in the ER [7], [19], [20], [34]. For the reasons stated above it is of great interest to investigate the spatial organization of the plant sterol pathway, by defining the subcellular localization of key enzymes.

In this report we describe the functional localization of Δ^7^-sterol-C_5_-desaturase (STE1/DWARF7), Δ^5,7^-sterol-Δ^7^-reductase (DWARF5) and Δ^24^-sterol-Δ^24^-reductase (DIM/DWARF1), three enzymes involved in the late steps of sitosterol biosynthesis, in *Arabidopsis thaliana*. These three enzymes constitute the last biosynthetic segment of the post-squalene sterol pathway where the tetracyclic moiety bearing a C_7_–C_8_ double bond undergoes a C_5_–C_6_ desaturation then a Δ^7^-reduction, to yield Δ^5^-sterols, the pathway end-products in wild-type *Arabidopsis thaliana* [35]. To this mandatory biosynthetic sequence Δ^7^→Δ^5,7^→Δ^5^ (Figure 1) is associated the reduction of the Δ^24^ double bond in the side chain of *eg* 24-methylene cholesterol and isofucosterol [13], [20], [35]. Plants and other eukaryotic organisms share the same biosynthetic segment, with the notable exception of yeast which does not have a sterol-Δ^7^-reductase and therefore accumulate ergosterol, a Δ^5,7^-sterol, as the major pathway end-product. We fused STE1/DWARF7, DWARF5 and DIM/DWARF1 with the yellow fluorescent protein (YFP), used genetic complementation of the corresponding yeast deficient mutants (*erg3*, deficient in the yeast Δ^7^-sterol-C_5_-desaturase; *erg4*, deficient in the yeast Δ^24^-sterol-Δ^24^-reductase) or expression of DWARF5 in wild-type yeast to demonstrate that the fusion proteins were fully functional. We then genetically complemented Arabidopsis mutant lines lacking the corresponding enzyme activity due to induced genomic mutations (*ste1-1* and *dwarf5-2*) or to a T-DNA insertion (*dim*). This clearly demonstrated that the fusion enzymes were active *in planta* and allowed detailed localization studies.

## Results and Discussion

### Yeast functional complementation

The coding regions of *STE1*, *DWARF5* and *DIM* from Arabidopsis were cloned as C-terminal translational fusions with the YFP reporter. In order to test whether the engineered enzymes were catalytically active these constructs were transformed into the appropriate yeast strains: STE1-YFP in *erg3*, DWARF5-YFP in a wild-type yeast, and DIM-YFP in *erg4*. It is well established that the enzymes involved in the late steps of the post-squalene sterol biosynthetic segment are highly conserved among plants, animals and fungi [20]; it has previously been shown that this allowed efficient reciprocal genetic complementation[35]–[37]. For instance Gachotte *et al*. [12] and Husselstein *et al*. [38] reported the characterization of the *ste1-1 Arabidopsis thaliana* mutant line that carries a T114I mutation in the Δ^7^-sterol-C_5_-desaturase enzyme, using the expression of STE1 in the *erg3* yeast mutant (Figure 1). We show here using the same *erg3* yeast strain that the fusion protein produced from of the *GAL1-10::STE1-YFP* construct is catalytically active. In fact, the *erg3* strain is unable to complete the ergosterol biosynthesis and accumulates episterol (Figure 1; Table 1) whereas the ergosterol biosynthetic pathway is partially restored after transformation of *erg3* with the *GAL1-10::STE1-YFP* fusion construct (Figure 1; Table 1).

**Table 1:** Sterol composition of wild type (W303) and mutant (*erg3* and *erg4*) yeast strains expressing the corresponding YFP-fused proteins compared to the non-transformed strains.

sterol (% of total)	W303	W303::*DWARF5YFP*	*erg3*	*erg3::STE1-YFP*	*erg4*	*erg4::DIM-YFP*
lanosterol (A)	5	3	8	8	3	5
4,4-dimethylzymosterol	3	2	4	3	6	4
episterol (B)	1	—	77	62	—	—
ergosta-7,22-dien-3β-ol (D)	—	—	10	8	—	2
ergosta-7,22,24(28)-trien-3β-ol (C)	—	—	1	1	—	1
ergosta-5-7-22-24(28)-tetraen-3β-ol (E)	3	2	—	3	91	82
ergosterol (F)	88	68	—	15	—	6
brassicasterol (G)	—	11	—	—	—	—
campesterol (H)	—	14	—	—	—	—

One representative analysis out of three independent experiments is shown. The letters in parentheses refers to Figure 1.- = compound not detected.

Similarly, the *erg4* mutant was complemented with the fusion protein produced from the *GAL1-10::DIM-YFP* construct, since *DIM* encodes the corresponding plant Δ^24^-sterol-Δ^24^-reductase (Figure 1). In the *erg4* strain, ergosterol is completely replaced by ergosta-5,7,22,24-tetraen-3β-ol (Figure 1; Table 1; [39]). The induction of the expression of the *GAL1-10::DIM-YFP* clearly re-established the biosynthesis of ergosterol in the mutant yeast (Table 1), demonstrating thus the catalytic activity of the engineered protein.

The wild-type W303-B1 yeast strain was used to assess the functionality of the DWARF5-YFP fusion protein encoding a Δ^5,7^-sterol-Δ^7^-reductase. Ergosterol as a Δ^5,7^-diene may be a substrate for the engineered Δ^5,7^-sterol-Δ^7^-reductase [40]. Our results show the reduction of the C-7(8) double bond of Δ^5,7^-sterols in yeast: the accumulation of brassicasterol and campesterol upon expression of the *GAL1-10::DWARF5-YFP* verifies DWARF5-YFP as a functional enzyme.

In conclusion, the expression of the fusion constructs in yeast and the detection of the expected sterol profiles confirmed that all three fusion enzymes were catalytically active.

### Plant functional complementation

**Figure 2:**
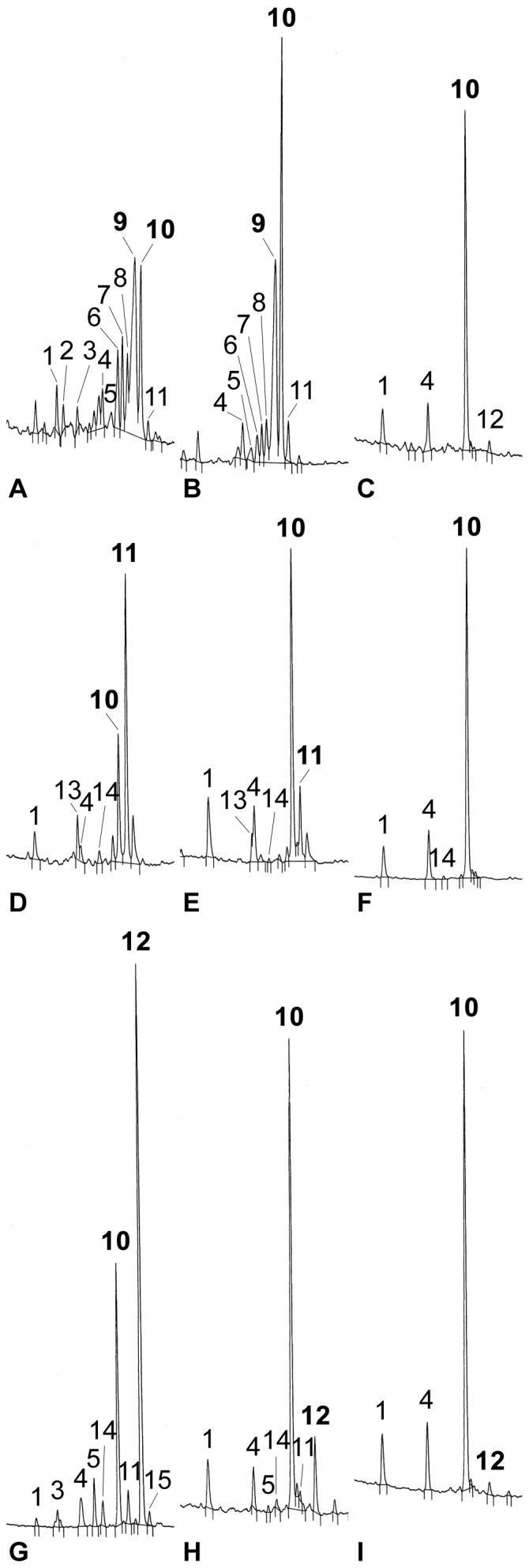
Genetic complementation of *ste1-1*, *dwarf5-2* and *dim* sterol biosynthetic mutants expressing *STE1-YFP*, *DWARF5-YFP*, and *DIM-YFP* cDNAs, respectively. GC-FID chromatograms of steryl acetates are shown. (A) *dwarf5-2* mutant; (B) *dwarf5-2*/*DWARF5-YFP* partially complemented mutant; (C) *dwarf5-2*/*DWARF5-YFP* fully complemented mutant. (D) *dim* mutant; (E), *dim*/*DIM-YFP* partially complemented mutant; (F) *dim*/*DIM-YFP* fully complemented mutant. (G) *ste1-1* mutant; (H), *ste1-1*/*STE1-YFP* partially complemented mutant; (I) *ste1-1*/*STE1-YFP* fully complemented mutant. Sterol peaks identified by their retention time and confirmed by GC-MS (prominent mass fragments not shown here) are: 1, cholesterol; 2, Δ^5,7^-cholesterol; 3, Δ^7^-cholesterol; 4, campesterol; 5, Δ^7^-campesterol; 6, Δ^5,7^-campesterol; 7, Δ^5,7^-stigmasterol; 8, Δ^8^-sitosterol; 9, Δ^5,7^-sitosterol; 10, sitosterol; 11, isofucosterol; 12, Δ^7^-sitosterol; 13, 24-methylene cholesterol; 14, stigmasterol; 15, Δ^7^-avenasterol. Full complementation of *dwarf5-2*, *dim* and *ste1-1* results in the accumulation of sitosterol (10) instead of Δ^5,7^-sitosterol (9), isofucosterol (11) and Δ^7^-sitosterol (12), respectively. The relevant peaks in each complementation are labelled in bold in the relevant panels.

Having demonstrated that the three YFP fusion enzymes were active in yeast, we transformed the *ste1-1*, *dwarf5-2* and *dim Arabidopsis* mutant lines with the corresponding fusion enzymes. The *ste1-1* and *dwarf5-2* mutants bear point mutations in the encoded enzymes (*i.e.* T114I in STE1, stop at 298 in DWARF5) and *diminuto/dwarf1 (dim)* is a T-DNA insertion mutant. The *dwarf5-2* mutation leads to the accumulation of Δ^5,7^-sterols, particularly Δ^5,7^-sitosterol, and to a lack of brassinosteroids as a consequence of a depletion in pathway end-products campesterol and sitosterol (Figure 2A; Table 2). For this reason, the *dwarf5-2* plants display a clear phenotype characterized by short height, short internodes, increased number of inflorescences, dark green round leaves, and a slow growth rate, compared to the wild type [10]. The *dim* mutant plants, similarly to the *dwarf5-2*, show a dwarf phenotype due to the deficiency in brassinosteroids, this being caused by a block in the Δ^24^-sterol-Δ^24^-reductase activity. Compared to the wild type, the *dim* plants show a reduced level of campesterol and sitosterol associated to an increase of 24-methylene cholesterol and isofucosterol, their respective metabolic precursors and substrates of DIM (Figure 2D; Table 2; [13]). Normally, plants only accumulate isofucosterol during early embryogenesis and in flower buds [41]. Similarly to the *dwarf5-2*, the *dim* seedlings have also short hypocotyls, petioles, and roots. Leaves of *dim* are round, curly, and dark green in color. In adult flowering plants, *dim* shows extremely short inflorescences with small flowers and severely reduced fertility [42]. In contrast, the *ste1-1* mutant plants show a morphological phenotype closely similar to the wild-type. These plants are impaired in the Δ^7^-sterol-C_5_-desaturation step [12], [38] and therefore accumulate Δ^7^-sterols (Δ^7^-campesterol and Δ^7^-sitosterol). Since *ste1-1* is a weak allele, homozygote *ste1-1* plants are able to synthesize about 30% of the pathway end-products Δ^5^-sterols (Figure 2G; Table 2) without compromising brassinosteroid biosynthesis [12], [38].

**Table 2:** Sterol composition of wild type and *dwarf5-2* (*d5*), *dim* (*d1*) and *ste1-1* (*s1*) Arabidopsis mutant plants.

sterol (% of total)	wild type	*d5*	*d5::DWARF5-YFP* part. compl.	*d5::DWARF5-YFP* fully compl.	*d1*	*d1::DIM-YFP* part. compl.	*d1::DIM-YFP* fully compl.	*s1*	*s1::STE1-YFP* part. compl.	*s1::STE1-YFP* fully compl.
Δ^8^-sitosterol (8)		8.2	5.0	—	—	—	—	—	—	—
Δ^7^-cholesterol (3)	2.3	2.5	—	—	—	—	—	1.0	—	—
Δ^7^-campesterol (5)		1.7	3.5	—	—	—	—	4.4	0.5	—
Δ^7^-avenasterol (15)		—	—	—	—	—	—	1.4	tr	—
Δ^7^-sitosterol (12)		tr	tr	1.5	—	—	—	58.5	10.3	3.3
Δ^5,7^-cholesterol (2)		2.5	—	—	—	—	—	—	—	—
Δ^5,7^-campesterol (6)		8.6	3.2	—	—	—	—	—	—	—
Δ^5,7^-sitosterol (9)		41.6	28.2	—	—	—	—	—	—	—
Δ^5,7^-stigmasterol (7)		8.2	3.8	—	—	—	—	—	—	—
cholesterol (1)	3.8	3.7	tr	8.8	6.5	4.6	6.2	0.1	6.2	2.8
24-methylene cholesterol (13)		—	—	—	7.7	4.2	tr	—	—	—
brassicasterol (16)	1.4									
campesterol (4)	16.2	3.0	2.6	12.9	2.0	11.7	16.3	4.3	7.0	17.5
isofucosterol (11)	4.9	1.7	3.8	—	56.6	15.5	tr	3.1	3.0	tr
sitosterol (10)	67.2	18.3	49.9	76.8	25.8	63.0	77.0	24.7	72.5	76.4
stigmasterol (14)	4.2	—	—	—	1.4	1.0	0.5	2.5	0.5	tr

The values refer to the partially and fully complemented plants compared to the not complemented. The numbers in parentheses refers to Figure 1 and Figure 2. tr = trace amount; - = compound not detected. Accurate sterol nomenclature can be found at IUPAC http://www.iupac.org.

**Figure 3:**
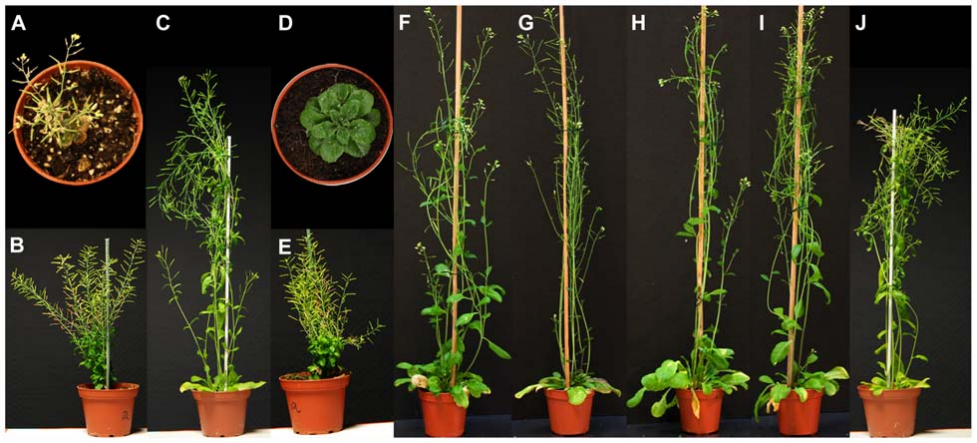
Phenotype of *dwarf5-2*, *dim* and *ste1-1* mutants complemented with *DWARF5-YFP*, *DIM-YFP* and *STE1-YFP*, respectively. (A, D, G) *dwarf5-2*, *dim* and *ste1-1* (sterol profiles given in Figure 2). (B, E, H) *dwarf5-2, dim and ste1.1* partial complemented. (C, F, I) *dwarf5-2*, *dim* and *ste1-1*fully complemented. (J) Wild-type (sterol profile given in Supplemental Figure 2).

C-terminal *YFP* fused constructs of *DWARF5*, *DIM* and *STE1* coding sequences were stably transformed into the corresponding *dwarf5-2*, *dim*, *and ste1-1* mutants. Primary transformants selected for their resistance to kanamycin were selfed to generate T2 then T3 generations. The success of the transformation and the efficiency of the generated constructs were revealed by lines that were fully complemented based on the morphology of individuals as shown for *dwarf-5* (Figure 3), and on sterol profiles for all three complemented *dwarf5-2*, *dim*, *and ste1-1* lines (Figure 2; Table 2). In the case of *dwarf5-2* and *dim* mutants, *dwarf5-2::DWARF5-YFP* and *dim::DIM-YFP* transgenic lines fall clearly into two categories according to their semi-dwarf phenotype indicating partial complementation, or to their wild-type phenotype indicating full complementation, when compared to the parental lines (Figure 3).

The integrity of the three YFP-fusion proteins in the transgenic plants was assessed by immunoblot analysis using an antibody directed towards the YFP-protein. This showed the accumulation of products with the expected molecular size for the DIM-YFP and DWARF5-YFP fusions. The STE1-YFP fusion protein was not detected nor was a degradation product (Supplemental Figure 1). Probably the abundance of the STE1-YFP protein was below the detection limit of the antibody.

The sterol profile established by GC-FID and confirmed by GC-MS for several individual plants from the T2 and T3 generations demonstrated the correct *in planta* enzyme activity for all fusion proteins: indeed, partial (Figure 2B, 2E, and 2H; Table 2) or full (Figure 2C, 2F, and 2I; Table 2) reversion of the sterol composition to wild-type was detected in all cases (Figure2; Supplemental Figure 2). In addition, the level of morphogenetic complementation and metabolic complementation were completely coincident. The fact that both partial and fully complemented plants were observed for each mutant line shows that the expression of the YFP fusion proteins has been performed in the correct mutant backgrounds.

The partial restoration of sitosterol biosynthesis observed in *ste1-1* mutants expressing the STE1-YFP fusion protein is in good agreement with the results reported by Husselstein *et al*. [38] who found the *ste1-1* plants showing a sitosterol recovery of about 50% when compared to wild-type, after transformation with a 35S::*STE1* construct. In conclusion, the observations of fully or partially complemented plants shows that all three C-terminal YFP fused enzymes are catalytically active *in planta*. The full complementation result in a sterol profile and content closely identical to that of the wild-type.

### Subcellular localization

The enzymes investigated in this work were chosen due to their implication in the late steps of sitosterol biosynthesis. Sterol metabolism and transport mechanisms have been investigated and documented in animals [43], [44] and to some extent in fungi with respect to cellular transport [45]. In plants, still little is known about the regulation of sterol biosynthesis and transports. Moreover, a detailed subcellular localization analysis of the late sterol biosynthetic enzymes is lacking. In order to establish the localization of STE1, DWARF5 and DIM, an *in silico* investigation was performed on their peptide sequences followed by extensive experimental investigations using confocal microscopy.

The *in silico* analysis made with the Predotar software based on the presence of signal peptides at N-termini of the protein sequences, predicted DWARF5 to be localized to the ER with a probability of 90% (Supplemental Table 1). In contrast DIM and STE1 were predicted to be non-ER proteins with a probability of 94% and 98%, respectively. These results are in agreement with the predictions obtained with Signal P, a protein sequence evaluation software, which found a significant probability of the presence of a signal peptide only in DWARF5 (Supplemental Table 1).

To experimentally assess whether STE1, DWARF5 and DIM were localized to the ER or to other compartments, we used confocal microscopy to analyse cells from the lower epidermal layer of leaves detached from functionally complemented *dwarf5-2::DWARF5-YFP*, *dim::DIM-YFP* and *ste1-1::STE1-YFP* plants.

**Figure 4:**
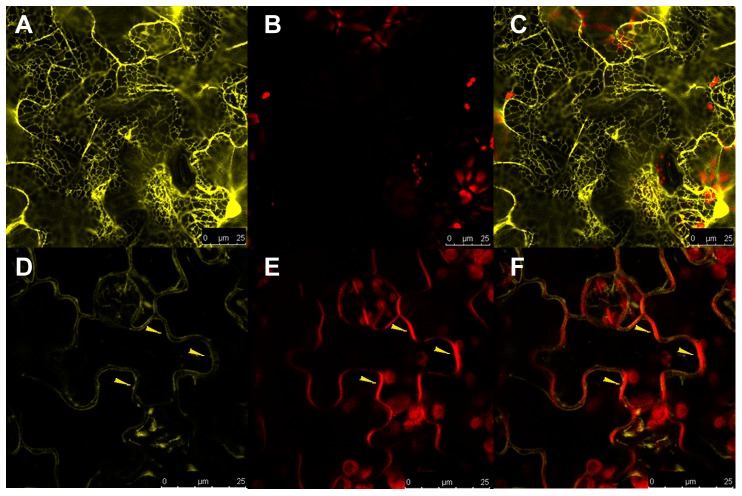
Subcellular localization of DWARF5-YFP protein in Arabidopsis *dwarf5-2::DWARF5-YFP* plants. (A) Confocal images of leaves showing protein distribution in the ER and (D) to the periphery of the cell (yellow arrow). (B) Chlorophyll autofluorescence. (E) In red is shown the chlorophyll autofluorescence combined with the FM4-64 fluorescence localized to the PM. The overlay images show (C) the complete separation of red and yellow signal and (F) the co-localization of DWARF5-YFP and FM4-64 indicating the PM association of DWARF5. Scale bars = 25 µm.

**Figure 5:**
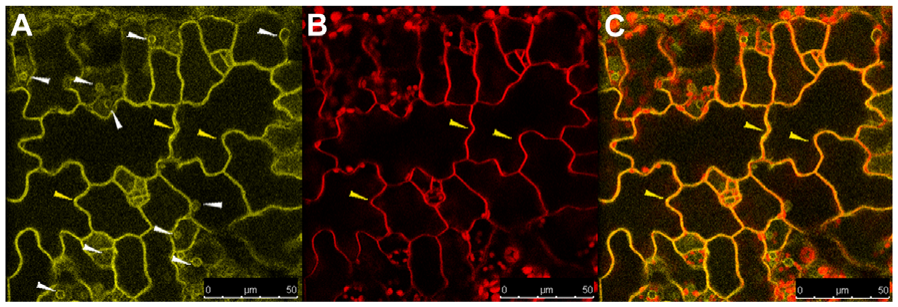
Subcellular localization of DIM-YFP protein in Arabidopsis *dim::DIM-YFP* plants. (A) The DIM-YFP signal is localized to structures surrounding the nuclei resembling ER (white arrow) and to the cell periphery (yellow arrow). (B) In red is shown the chlorophyll autofluorescence combined with the FM4-64 fluorescence localized to the PM. (C) The overlay image shows the co-localization of DIM-YFP and FM4-64 suggesting the PM association of DIM. Scale bars = 50 µm.

In accordance with the *in silico* prediction (Supplemental Table 1), the DWARF5-YFP fusion protein showed a clear reticulate structure in confocal observation corresponding to a typical ER localization (Figure 4A). This ER localization is consistent with observations reported in the case of the orthologous enzyme 7-dehydrocholesterol reductase (7-DHCR) in mammalian cells [46]. Moreover we observed a clear signal at the periphery of the epidermal cell (Figure 4). An ER-like pattern was seen for the DIM-YFP fusion protein (Figure 5A; Supplemental Figure 3A), which also showed a clear signal in the peripheral area (Figure 5A, 5D). In both cases the peripheral signal suggests that these proteins are synthesized in the endomembrane system for delivery to the PM [47].

To further investigate the association of these two proteins to the PM, we stained the leaves of transgenic *dwarf5-2* and *dim* plants expressing the DWARF5-YFP (Figure 4E) and DIM-YFP (Figure 5B and 5C), respectively, with the membrane dye FM4-64.

Interestingly, the human 24-DHCR (SELADIN1), as well as the yeast ERG4p, two orthologs of the plant DIM, have been both localized mainly to the ER [39], [48] but also to a lesser extent to Golgi complexes [48], indicating possible dual localizations for enzymes of this type. Previously, Klahre *et al*. [13] found a N-terminal GFP-DIM fusion protein localized in speckled structures in the cytoplasm of pollen tubes; this was interpreted as ER. Localization to the Golgi may indicate a possible translocation of this protein to the PM in secretory vesicles. This vesicular transport is known and has been well described in the case of yeast [45]. Moreover, the DIM localization in the PM can be interpreted as necessary for the fine tuning of the membrane properties by regulating the content of sitosterol (*i.e.* the ratio of isofucosterol to sitosterol) and campesterol and consequently the response to environmental changes [49]. In addition, DIM was already identified as a component of detergent resistant membranes (DRM) or so-called lipid rafts isolated from tobacco PM [4].

**Figure 6:**
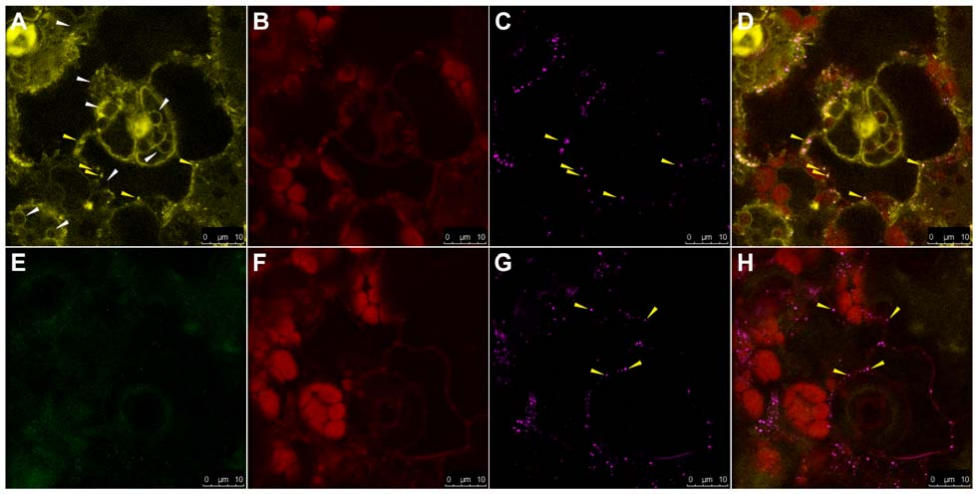
Subcellular localization of STE1-YFP protein in Arabidopsis *ste1-1::STE1-YFP* plants (panel A to D) and Nile Red staining of ste1-1 mutants (panel E to H). (A) STE1-YFP localization to structures resembling ER (white arrow) and LPs (yellow arrows). (B) and (F) chlorophyll autofluorescence. (C) and (G) LPs stained with Nile Red (yellow arrows). (D) Overlay image of (A), (B) and (C) showing the overlap of the Nile Red and YFP signal (yellow arrow) and the ER localization (white arrow) of STE1-YFP. (E) YFP signal absent in *ste1-1* mutant. (H) Overlay image of (E), (F) and (G) showing the distribution of LPs in cell of *ste1-1* plant. Scale bars = 10 µm.

In addition to DWARF5-YFP and DIM-YFP, the STE1-YFP expressed in transformed *ste1-1* mutant plants also displayed an ER-like pattern (Figure 6A; Supplemental Figure 3D). Surprisingly, for the STE1-YFP fusion we further observed an unexpected localization in small and round structures moving across the cytoplasm (Supplemental Movie 1). As the observed structures resembled LPs (Figure 6A, 6D), the STE1-YFP fusion localization was further investigated by incubating leaves from *ste1-1* mutant plants with Nile Red, a lipid specific dye staining cellular LPs. Results from Nile Red staining showed a pattern characterized by clear signals deriving from round bodies dispersed into the cytoplasm (Figure 6B, 6C, 6D, 6G, 6H). The localization of STE1-YFP was observed to overlap with Nile Red signals, indicating that STE1-YFP is localized to LPs as well as to ER (Figure 6D).

LPs consist of a phospholipid monolayer which sequesters the hydrophobic core of LPs from the cytosol. This has been well described in yeast. The core of the particles consists of triacylglycerols (TAG) surrounded by several layers of SE [50], [51]. The LPs represent a storage compartment for fatty acids and sterols in the form of conjugates, and appear to be involved in their translocation from ER to all membranes of the cells. The mechanism of sterol translocation between ER and LPs is not completely clear [52], although it has been recently demonstrated that the lipid droplets could originate from the ER [53] and are probably involved into a non-vesicular sterol transport to the PM [45].

Studies performed on a tobacco mutant overproducing sterols showed that the excess of sterols was converted into steryl esters (steryl plamitate, oleate, linoleate and linolenate) which were accumulated into LPs [24]. The storage and translocation mechanism would then be impossible without enzymes that mobilize these conjugates. In yeast, although the sterol synthesizing enzymes are mainly localized in the ER, ERG1, ERG27 and ERG6 enzymes show a dual localization on ER and LPs while ERG7 has been localized only on LPs [52]. In addition, four of the seven acyltransferases [32], one of the three lipases [33] and two of the three SE hydrolases identified until now [50] have been localized to LPs in yeast. Recently, a phospholipid-sterol acyltransferase (PSAT1) and a sterol-O-acyltransferase (ASAT1) have been identified in *Arabidopsis* where the enzyme activities were associated with microsomal membranes [27], [28], [54]. The esterification reaction and the major enzyme responsible for this process, namely, the PSAT1, was described but so far plant SE hydrolases have not been identified.

**Figure 7:**
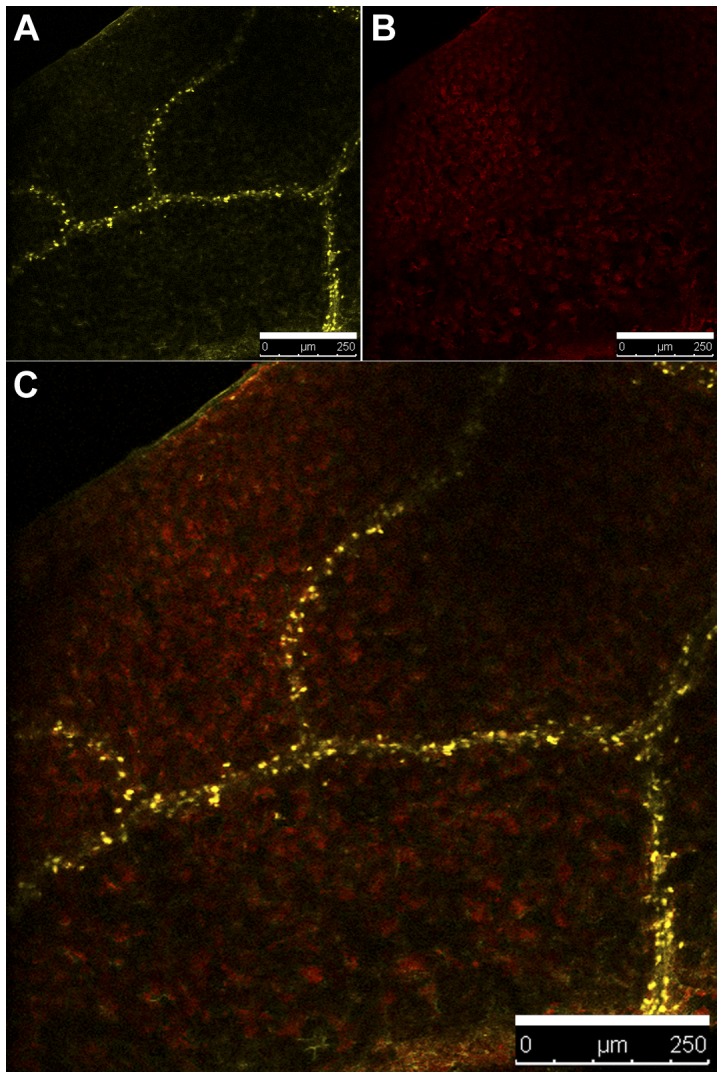
Subcellular localization of STE1-YFP protein in Arabidopsis *ste1-1::STE1-YFP* plants. (A) STE1-YFP fluorescence signal in leaf vascular tissue. (B) Chlorophyll autofluorescence. (C) Overlay image of (A) and (B). Scale bars = 250 µm.

The Δ^7^-sterol-C_5_-desaturase (STE1) displays a dual localization in the ER and the LPs in *Arabidopsis*, revealing an unexpected subcellular localization for this enzyme, and a possible yet unknown role of the LPs in in sterol biosynthesis. Interestingly, the link between sterol esterification and the activity of the Δ^7^-sterol-C_5_-desaturase has been shown in yeast where the disruption of the sterol acyltransferase genes *ARE1* and *ARE2* were followed by a decrease of *ERG3* expression [55]. This indicates that the absence of sterol esterification leads to a decrease in total intracellular sterols. It could assign a role for ERG3 in the storage process of sterol esters. Moreover, the examination of microarray data related to the expression of *STE1* in wild-type *Arabidopsis* (The Bio-Array Resource for Plant Biology http://www.bar.utoronto.ca/; [56]) revealed a high level of *STE1* expression in seeds. This indication also could be viewed as part of the lipid accumulation process in seeds. We further analyzed *ste1-1::STE1-YFP* plants by confocal microscopy and were able to detect strong signals along the vascular tissues from giant compartments which were then identified as LPs (Figure 7). Interestingly, it has been reported that the silencing of the major seed oleosin gene of Arabidopsis resulted in an aberrant phenotype of embryo cells that contain larger oil bodies than those found in the wild-type [57]. Changes in the size of oil bodies caused disruption of storage organelles, altering accumulation of lipids and proteins and causing delay in germination. The vascular localization could suggest a systemic lipid transport in plant, probably associated with lipoproteins as for instance oleosins [58] and steroleosin [59]. A mechanism of that type is well known in the case of mammalian cholesterol homeostasis, implicating in particular an extracellular transport of cholesterol through the LDL/LDL receptor machinery [60].

In conclusion we have shown that C-terminal YFP-fusion proteins of DWARF5, DIM and STE1 were found to localize to the endoplasmic reticulum. Surprisingly, Δ^24^-sterol-Δ^24^-reductase (DIM) was further localized to the plasma membrane, whereas Δ^7^-sterol-C_5_-desaturase (STE1) was also clearly detected in lipid particles. We suggest that the partial and/or full complementation of all three biochemical mutants, that is, a partial and/or full restoration of a wild-type sterol profile and morphology upon expression of the YFP-fusions, indicate a relevant subcellular localization of the engineered enzymes. In fact, the identical confocal images observed whether the complementation was partial or full rule out a hypothetical mislocalization of the accumulated YFP-fusions. Therefore, the different localization patterns observed for the three different enzymes support their correct subcellular localization. Together these findings raise new challenging questions about the spatial organization of sterol biosynthesis in plants and the implication of this localization in cellular lipid homeostasis.

## Methods

### Plant lines and culture conditions

*Arabidopsis thaliana* plants from the ecotype Enkheim 2 (wild-type En-2 and mutant line *dwarf 5-2*; [10]), Columbia-*glabrous 1* (wild-type Col-*gl1* and mutant line *ste1-1*; [12]) and Columbia-0 (wild-type Col-*0/C24* and mutant line *dim*; [13]) were grown in greenhouse conditions with a light/dark regime of 16/8 hours and a temperature setting of 18 °C during the light period and 15 °C during the dark period. Four-week old seedlings were transferred to individual pots and grown to flowering stage. Plant transformations with the relevant constructs were performed using the *Agrobacterium tumefaciens* floral dip method [61]. Primary transformants were grown for two more generations for further analysis. One month-old rosette leaves were sampled from 5 plants of a given genotype in three independent experiments and immediately frozen in liquid nitrogen, freeze dried (ScanVac® model CoolSafe Pro 100-4, ScanLaf A/S, Denmark) and stored at −80 °C prior to sterol analysis. One-week old seedlings, germinated on Murashige and Skoog medium (0,4% MS, 2% sucrose, 1% agar) were used for confocal microscopy observations. A minimum of three transgenic lines for each type of genetic complementation experiment were considered in the present study.

### Yeast strains and growth conditions

The wild-type yeast strain Saccharomyces cerevisiae W303-1A (*MAT a; ura3-52; trp1Δ 2; leu2-3,112; his3-11; ade2-1; can1-100*) and the mutant strains *erg3* (*ORF YLR056w: BY4741; MATa; his3*Δ*1; leu2*Δ*0; met15*Δ*0; ura3*Δ*0; YLR056w::kanMX4*) and *erg4* (*ORF YGL012w: BY4742; MATα; his3*Δ*1; leu2*Δ*0; lys2*Δ*0; ura3*Δ*0; YGL012w::kanMX4*) were obtained from the European Saccharomyces Cerevisiae Archives for Functional Analysis (EUROSCARF). The yeast strains were grown aerobically at 30 °C either in YPD containing 2% glucose (Merck), 1% Yeast Extract (Oxoid) and 2% peptone (Oxoid) or in synthetic minimal (SM) media containing 0.67% Yeast Nitrogen Base w/o amino acids and w/o ammonium sulphate (Remel®), 0.2% yeast synthetic Drop-out medium supplements (Sigma-Aldrich, Steinheim, Germany) excluding uracil (W303 and *erg3*) or histidine (*erg4*) and 2% bactoagar (Difco®). The SM media contained either 2% glucose or galactose (Sigma-Aldrich®) as carbon source. Milli-Q water was used (18 MΩ, Millipore®, Billerica, USA).

The strains bearing the YFP fused gene constructs integrated into the genome were generated by the lithium acetate method [62]. Liquid cultures in selective synthetic media containing 2% galactose were performed at 30 °C in a shaking incubator (Thermo-Forma Scientific, Lytzenlab A/S, Denmark) at 220 rpm in order to activate the YFP fused gene transcription. Liquid cultures in presence of 2% glucose were produced as control. Yeast pellets were collected by centrifugation at 4000×g (Laborzentrifugen, Sigma®) of 50 ml of three days-old liquid culture were frozen in liquid nitrogen, freeze dried and stored at −80 °C prior to sterol analysis.

### ENZYME::YFP constructs

The cDNAs encoding DWARF5 (At1g50430), STE1 (At3g02580) and DIM (At3g19820) were obtained from the Arabidopsis Biological Research Centre (ABRC, Ohio, US). Translational fusions of *DWARF5*, *STE1* and *DIM* to the *YFP* reporter were generated by cloning the corresponding ORFs into a pLIFE001 vector, a modified version of pCAMBIA 2300 plant expression vector suitable for User cloning [63]. The pLife001 vector contains the YFP coding sequence at the 3′ of the MCS where its expression is under the control of the CaMV 35S promoter. Cloning was performed by uracil-excision based cloning technique as described [63]. For this purpose cDNA sequences were PCR-amplified with specific uracil containing primers (Supplemental Table 2) by using the improved Pfu X7 polymerase [64].

In order to generate the translational fusions necessary for the yeast complementation experiments, the *cDNA-YFP* sequences were amplified from the plant expression vectors with appropriate Gateway® cloning primers (Supplemental Table 3). Amplified sequences were recombined first into pDONR211 (Invitrogen®) and then into episomal yeast expression vectors carrying the desirable selection marker (HIS+ or URA+) under the control of the *GAL1-10* galactose inducible promoter [65]. The obtained constructs *GAL1-10::DWARF5-YFP* and *GAL1-10::STE1-YFP* were cloned into pMP2360 (URA3+) and *GAL1-10::DIM-YFP* into pMP1965 (HIS3+), which are modified Gateway versions of pRS426-GAL and pRS423-GAL, respectively [66] (kindly provided by Dr. Rosa Lopez, University of Copenhagen, DK).

*Escherichia coli* strain DH10β was used for DNA cloning. Standard cloning procedures were followed [67].

### Sterol extraction and analysis

#### Lipid extraction

The freeze-dried plant tissues and yeast pellets (50 mg±2 mg) were transferred into 50 ml tubes, mixed with 15 ml 6% (w/v) KOH in methanol and homogenized using an Ultra Turrax® homogenizer. Saponification was carried out at 70 °C for 2 hours. Subsequently 5 ml Milli-Q water and 20 ml pentane were added to each tube and vortexed twice for 30 sec. The tubes were subsequently centrifuged at 4000 *g* for 5 minutes (Laborzentrifugen, Sigma®) at room temperature, and the organic fractions were transferred to Erlenmeyer flasks. The extraction was repeated twice with 20 ml pentane and the organic phases were pooled. The combined extracts were evaporated to dryness in a rotary evaporator at 30°C (Rotavapor® model R-215, Büchi, Switzerland). The residues were resuspended in 1 ml pentane and transferred to a 1.5 ml glass GC vials (Mikrolab A/S, Denmark). The samples were then evaporated under a stream of Nitrogen gas and stored in argon atmosphere at −80 °C prior to analysis. Pentane and methanol were GC grade (Fluka®). Potassium hydroxide pellets (for analysis) were from Merck® (Damstadt, Germany).

### Sterols purification from plant extracts

Plant or yeast sterols were purified by TLC (60 F254 silica plates, Merck, Darmstadt, Germany), with two runs in dichloromethane. 7-dehydrocholesterol (Purity>98%) was purchased from Sigma-Aldrich and used as standard. After the TLC run, the bands in the samples lanes showing the same retention factor (R_f_) as the standard, resolved by UV-B light exposure, were scraped off the plate and eluted with pentane. The eluted fractions were evaporate to dryness, dissolved in a small volume of pentane, transferred to a 1.5 ml glass GC vials, dried again and stored in argon atmosphere at -80 °C. The vials containing sterols were processed for characterization by GC-MS and GC-FID.

### GC-MS and GC-FID characterization

The yeast and plant sterol fractions were resuspended in 100 µl toluene and derivatized for GC assays by adding 40 µl of acetic anhydride (Sigma) and 25 µl of pyridine (Fluka). Acetylation was carried out for 1 hour at 70 °C. The samples were than evaporated to dryness and resuspended in 500 µl of hexane. Sterols were separated and identified by GC-FID (gas chromatography–flame ionization detection) using a Varian 8400 gas chromatograph equipped with a DB-5 capillary column, wall coated, open, and tubular; 30 m; 0.25 mm film thickness, 32 mm i.d.; Agilent J&W, USA) using H_2_ as carrier gas (2 ml/min). The GC oven program included a fast increase from 60 °C to 220 °C (30 °C/min) and a slow increase from 220 °C to 300 °C (2 °C/min). Data from the detector were monitored with the VARIAN STAR computer program (Varian, Walnut Creek, CA, USA). Sterol structures were confirmed by GC-MS (Agilent 6890 gas chromatograph and 5973 mass analyzer) equipped with a HP-5MS glass capillary column (wall coated, open, and tubular; 30 m; 0.25 mm film thickness, 32 mm i.d.; Agilent J&W, USA) using hydrogen as carrier gas.

### Plant subcellular localization

The computational predictions were performed on the internet website interfaces provided by each prediction program. The prediction programs used in this study are Predotar (ver 1.03; INRA/CNRS/UEVE, FR) (http://urgi.versailles.inra.fr/predotar/predotar.html) [68] and SignalP (ver 4.0; CBS, DK) (http://www.cbs.dtu.dk/services/SignalP/) [69]. Experimental subcellular localization of STE1-YFP, DWARF5-YFP and DIM-YFP was carried out by confocal observation of leaf cells from one-week old seedlings of the respective transformed *ste1-1*, *dwarf5-2*, and *dim* mutants showing complementation. FM4-64 (T-3166, Invitrogen®) was used for co-localization in the plasma membrane (PM) while Nile Red (N-3013, Sigma Aldrich) was used for co-localisation in the lipid particles (LPs).

Images were captured with a confocal microscope Leica SP5X equipped with a HCX PL APO lambda blue 20×/0,7 NA water immersion objective. YFP and FM4-64 were excited at 514 nm. Emission was collected at 505–565 nm for YFP and 593–682 nm for chloroplast autofluorescence and FM4-64 staining. Nile red was excited at 488 nm and emission was collected at 587–616 nm. Incubation time for all dyes (working solution 10 µg.mL^−1^) was 10 min. To exclude the localization in Golgi apparatus, leaves were submerged into a solution of Brefeldin A (10 µg.mL^−1^) and incubated at 25 °C for the times given [70]. Confocal images were analysed using the LAS AF software (Leica).

## Supporting Information

Figure S1 **Western blot analysis of wild type Arabidopsis and** ***dwarf5-2*** **,** ***ste1-1*** **and** ***dim*** **mutant plants expressing the corresponding YFP-fused proteins.** The figure shows the accumulation of DWARF5-YFP (∼79 KDa) and DIM-YFP (∼92 KDa). STE1-YFP was not detected probably due to the relative low abundance in the analysed tissues. Protein extracts were prepared by homogenization of frozen tissues in sample buffer. Equal amounts of total proteins were loaded on a 12% acrylamide gel and analysed by western blot using anti-GFP antibodies. A protein extract form plants carrying a 35S::*YFP* construct was used as control.
(TIF)

Figure S2 **Sterol profile and composition of an Arabidopsis wild type plant.** Sterol peaks identified by their retention time and confirmed by GC-MS (prominent mass fragments not shown here) are: 1, cholesterol; 3, Δ7-cholesterol; 4, campesterol; 10, sitosterol; 11, isofucosterol; 14, stigmasterol; 16, brassicasterol. In bold the more abundant sterol.
(TIF)

Figure S3 **Subcellular localization of DIM-YFP and STE1-YFP proteins in Arabidopsis.** Confocal images of leaves showing localization of (A) DIM-YFP and (D) STE1-YFP in the cell. (B, E) Chlorophyll autofluorescence. (C, F) Overlay images of YFP and autofluorescence channels. Scale bars = 25 µm.
(TIF)

Table S1 **In silico prediction of subcellular localization (Predotar) and signal peptide presence (SignalP) for DWARF5, STE1 and DIM based on their amino acid sequences.**
(DOC)

Table S2 **Primers used to assembly the YFP fused constructs.**
(DOC)

Table S3 **Primers used for cloning of the YFP-fused constructs into inducible yeast expression vectors.** The YFP reverse primer is common for all the constructs being generated on the YFP 3′ sequence.
(DOC)

Movie S1 **Subcellular localization of STE1-YFP in Arabidopsis** ***ste1-1::STE1-YFP*** **plants.** The movie is showing the subcellular trafficking of LPs observed while collecting the YFP signal.
(MOV)
