# Supplementary figures and images for: Plant Sterol Metabolism. Δ7-Sterol-C5-Desaturase (STE1/DWARF7), Δ5,7-Sterol-Δ7-Reductase (DWARF5) and Δ24-Sterol-Δ24-Reductase (DIMINUTO/DWARF1) Show Multiple Subcellular Localizations in Arabidopsis thaliana (Heynh) L
Source: PLoS One. 2013 Feb 8;8(2):e56429. doi: 10.1371/journal.pone.0056429 (PMC3568079; doi:10.1371/journal.pone.0056429)

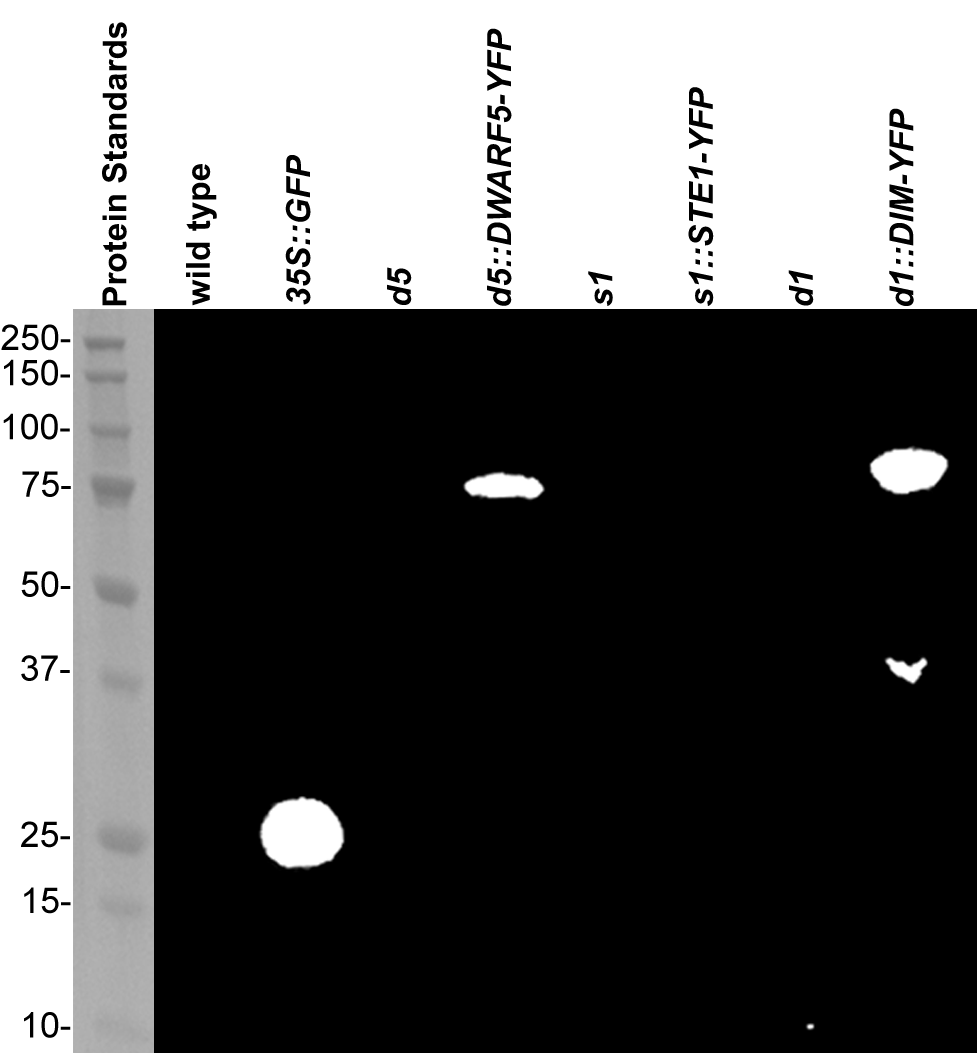

Supplement: Figure S1 — Western blot analysis of wild type Arabidopsis and dwarf5-2 , ste1-1 and dim mutant plants expressing the corresponding YFP-fused proteins. The figure shows the accumulation of DWARF5-YFP (∼79 KDa) and DIM-YFP (∼92 KDa). STE1-YFP was not detected probably due to the relative low abundance in the analysed tissues. Protein extracts were prepared by homogenization of frozen tissues in sample buffer. Equal amounts of total proteins were loaded on a 12% acrylamide gel and analysed by western blot using anti-GFP antibodies. A protein extract form plants carrying a 35S::YFP construct was used as control. (TIF) [file pone.0056429.s001.tif]

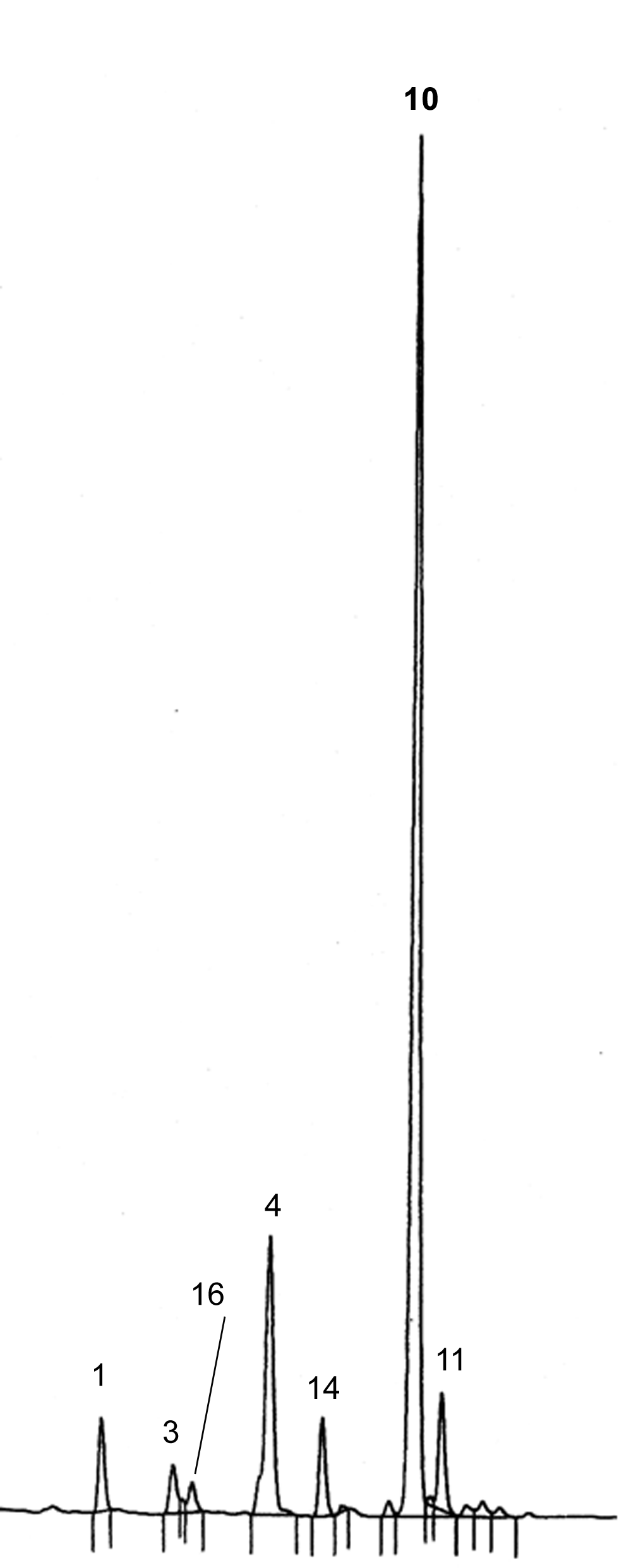

Supplement: Figure S2 — Sterol profile and composition of an Arabidopsis wild type plant. Sterol peaks identified by their retention time and confirmed by GC-MS (prominent mass fragments not shown here) are: 1, cholesterol; 3, Δ7-cholesterol; 4, campesterol; 10, sitosterol; 11, isofucosterol; 14, stigmasterol; 16, brassicasterol. In bold the more abundant sterol. (TIF) [file pone.0056429.s002.tif]

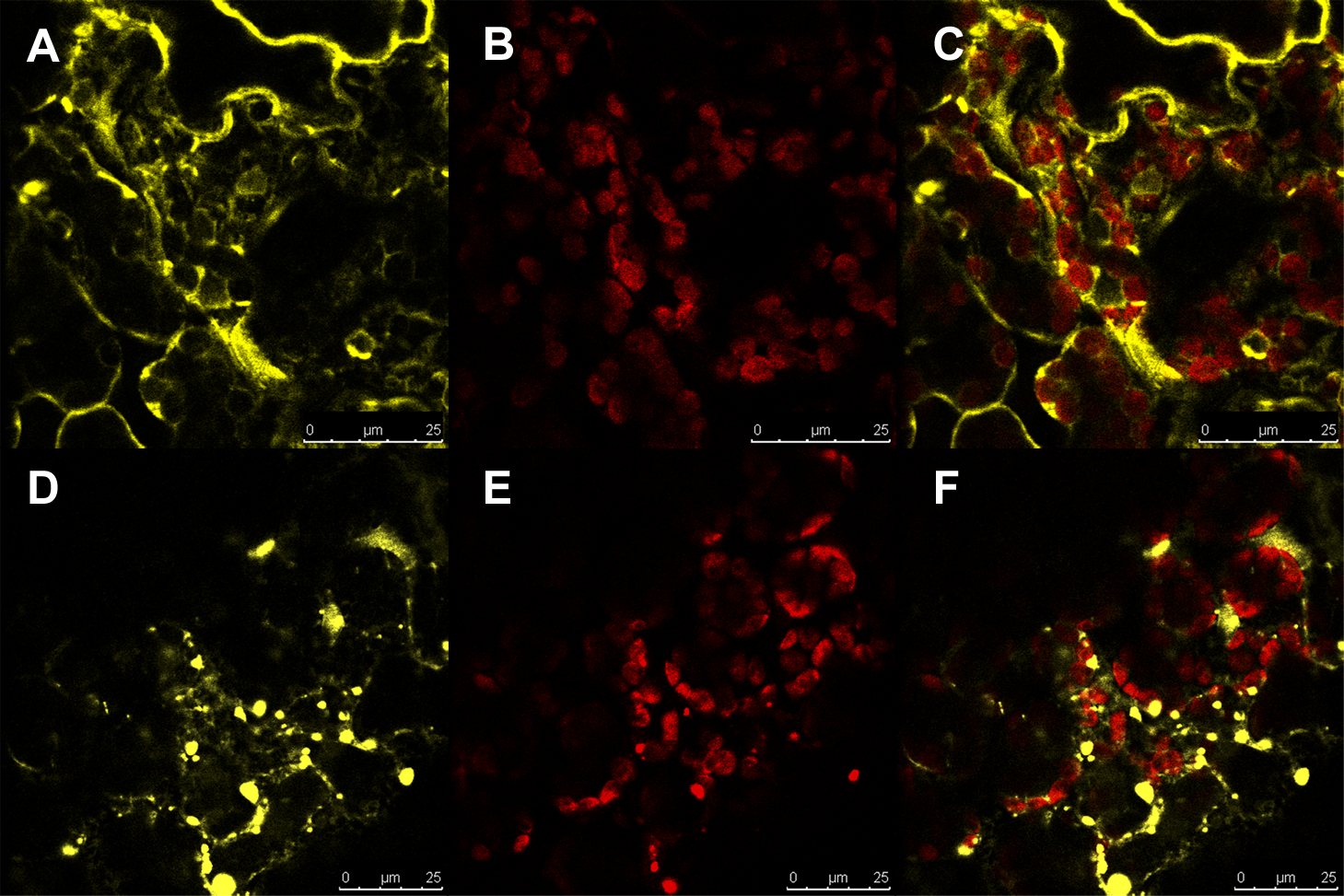

Supplement: Figure S3 — Subcellular localization of DIM-YFP and STE1-YFP proteins in Arabidopsis. Confocal images of leaves showing localization of (A) DIM-YFP and (D) STE1-YFP in the cell. (B, E) Chlorophyll autofluorescence. (C, F) Overlay images of YFP and autofluorescence channels. Scale bars = 25 µm. (TIF) [file pone.0056429.s003.tif]
