# Supplementary material for: Plant Sterol Metabolism. Δ7-Sterol-C5-Desaturase (STE1/DWARF7), Δ5,7-Sterol-Δ7-Reductase (DWARF5) and Δ24-Sterol-Δ24-Reductase (DIMINUTO/DWARF1) Show Multiple Subcellular Localizations in Arabidopsis thaliana (Heynh) L
Source: PLoS One. 2013 Feb 8;8(2):e56429. doi: 10.1371/journal.pone.0056429 (PMC3568079; doi:10.1371/journal.pone.0056429)
Supplement: Table S1 — In silico prediction of subcellular localization (Predotar) and signal peptide presence (SignalP) for DWARF5, STE1 and DIM based on their amino acid sequences. (DOC) [file pone.0056429.s004.doc]

**Table S1.** *In silico* prediction of subcellular localization (Predotar) and signal peptide presence (SignalP) for DWARF5, STE1 and DIM based on their amino acid sequences.

| **Protein** | **Software** | | | | | |
| --- | --- | --- | --- | --- | --- | --- |
|  | **Predotar** | | | | **SignalP** | |
|  | **Mitocondrial** | **Plastidial** | **ER** | **Elsewhere** | **Signal peptide** | **Signal anchor** |
| DWARF5 | 0.01 | 0.00 | **0.90** | 0.10 | 0.447 | 0.412 |
| STE1 | 0.01 | 0.01 | 0.00 | **0.98** | 0.000 | 0.848 |
| DIM | 0.05 | 0.00 | 0.01 | **0.94** | 0.001 | 0.997 |
