# Supplementary material for: Plant Sterol Metabolism. Δ7-Sterol-C5-Desaturase (STE1/DWARF7), Δ5,7-Sterol-Δ7-Reductase (DWARF5) and Δ24-Sterol-Δ24-Reductase (DIMINUTO/DWARF1) Show Multiple Subcellular Localizations in Arabidopsis thaliana (Heynh) L
Source: PLoS One. 2013 Feb 8;8(2):e56429. doi: 10.1371/journal.pone.0056429 (PMC3568079; doi:10.1371/journal.pone.0056429)
Supplement: Table S2 — Primers used to assembly the YFP fused constructs. (DOC) [file pone.0056429.s005.doc]

## Table S2. Primers used to assembly the YFP fused constructs.

| **cDNA** | **Primer** | **Sequence 5’ → 3’** |
| --- | --- | --- |
| *DWARF5* | Forward | GGC TTA AUA TGG CGG AGA CTG TAC ATT CTC |
|  | Reverse | GGT TTA AUC CAT AAA TTC CCG GAA TGA TCC TG |
| *STE1* | Forward | GGC TTA AUA TGG CGG CGG ATA ATG CTT ATC |
|  | Reverse | GGT TTA AUC CCT CTG CTT TCT TGA AGC TGT C |
| *DIM* | Forward | GGC TTA AUA TGT CGG ATC TTC AGA CAC C |
|  | Reverse | GGT TTA AUC CAT CTG CCT CGG CAT AAG CAG |
