# Supplementary material for: Plant Sterol Metabolism. Δ7-Sterol-C5-Desaturase (STE1/DWARF7), Δ5,7-Sterol-Δ7-Reductase (DWARF5) and Δ24-Sterol-Δ24-Reductase (DIMINUTO/DWARF1) Show Multiple Subcellular Localizations in Arabidopsis thaliana (Heynh) L
Source: PLoS One. 2013 Feb 8;8(2):e56429. doi: 10.1371/journal.pone.0056429 (PMC3568079; doi:10.1371/journal.pone.0056429)
Supplement: Table S3 — Primers used for cloning of the YFP-fused constructs into inducible yeast expression vectors. The YFP reverse primer is common for all the constructs being generated on the YFP 3′ sequence. (DOC) [file pone.0056429.s006.doc]

| **Construct** | **Primer** | **Sequence 5’ → 3’** |
| --- | --- | --- |
| *DWARF5-YFP* | Forward | GGG GAC AAG TTT GTA CAA AAA AGC AGG CTT AAT GGC GGC GGA TAA TGC TT |
| *STE1-YFP* | Forward | GGG GAC AAG TTT GTA CAA AAA AGC AGG CTT AAT GGC GGA GAC TGT ACA TT |
| *DIM-YFP* | Forward | GGG GAC AAG TTT GTA CAA AAA AGC AGG CTT AAT GTC GGA TCT TCA GAC AC |
| *YFP* | Reverse | GGG GAC CAC TTT GTA CAA GAA AGC TGG GTT TTA CTT GTA CAG CTC GTC CA |

Table S3. Primers used for cloning of the YFP-fused constructs into inducible yeast expression vectors**.** The YFP reverse primer is common for all the constructs being generated on the YFP 3’ sequence.
